# The Impact of Inappropriate Use Patterns on Sub-Lethal Antibiotic Exposure: A Multivariate Analysis on the Selection Risk of Resistant Mutants

**DOI:** 10.3390/antibiotics15060569

**Published:** 2026-06-03

**Authors:** Afet Arkut, Saime Uluçaylı, Hatice Sütçü, Mehmet Zeki Avcı

**Affiliations:** 1 Faculty of Health Sciences, Cyprus International University, via Mersin 10, Nicosia 99258, Turkey; aarkut@ciu.edu.tr (A.A.); hsutcu@ciu.edu.tr (H.S.); 2 Faculty of Economics and Administrative Sciences, Cyprus International University, via Mersin 10, Nicosia 99258, Turkey; 3 Faculty of Health Sciences, Cyprus Science University, via Mersin 10, Kyrenia 99300, Turkey; mehmetavci@csu.edu.tr

**Keywords:** antimicrobial resistance, nursing education, treatment non-adherence, sub-lethal dose

## Abstract

**Background**: Antimicrobial resistance (AMR) is an evolutionary crisis accelerated by inappropriate antibiotic use. While awareness studies often focus on descriptive knowledge, evidence-based predictive models identifying how cognitive deficits trigger treatment non-adherence are lacking. This study analyzed predictors of antibiotic non-adherence among university students using a multivariate behavioral-microbiological approach. **Methods**: A cross-sectional KAP (Knowledge, Attitude, and Practice) survey was conducted with 1044 students (Nursing: *n* = 620; non-healthcare: *n* = 424) in Northern Cyprus. A validated questionnaire assessed antimicrobial awareness. Multivariate logistic regression identified independent predictors (Adjusted Odds Ratio [AOR]) of treatment non-adherence. **Results**: Nursing students achieved significantly higher median scores in knowledge (9 vs. 7), attitude (10 vs. 8), and practice (9 vs. 7) compared to non-healthcare students (*p* < 0.001). Within the nursing cohort, a significant linear progression in KAP scores occurred from the 1st to 4th academic years (*p* < 0.05). The strongest independent predictor of antimicrobial non-adherence was inappropriate attitude (AOR: 2.100; 95% CI: 1.586–2.780; *p* < 0.001), followed by inadequate knowledge (AOR: 1.536; 95% CI: 1.160–2.033; *p* = 0.003). Conversely, nursing education was a significant protective factor (AOR: 0.669; *p* = 0.005). **Conclusions**: Antibiotic non-adherence is a complex psychological behavior management issue rather than merely an information deficit. Incorrect attitudes primarily drive sub-lethal dose exposure, theoretically increasing the epidemiological risk associated with resistant mutant selection. Future antimicrobial stewardship (AMS) programs must transcend traditional educational models and incorporate behavioral economics principles, such as digital nudging, to modify inappropriate attitudes.

## 1. Introduction

Antimicrobial resistance (AMR) is a major public health crisis that poses a multifaceted challenge to global health systems in light of current evidence. Global models of the bacterial AMR burden have shown that in 2019, there were approximately 4.95 million deaths associated with AMR and approximately 1.27 million deaths directly attributable to AMR; this data highlights that AMR is not merely a clinical treatment issue but also a structural threat requiring health policy and community-based prevention strategies [1,2]. Furthermore, projections suggest that if resistance trends are not brought under control, the global death toll could reach one person every three seconds by 2050. This substantial burden of mortality underscores the global public health priority of managing AMR trends [1,3]. The development of resistance at the microbiological level is accelerated by the accumulation of mutations in bacteria under selective pressure and the horizontal spread of resistance determinants via plasmids and other mobile genetic elements [4,5,6,7]. Therefore, the inappropriate use of antibiotics—such as their use for incorrect indications, premature discontinuation of treatment, or over-the-counter procurement—not only reduces the success of individual treatments but also facilitates the selection of resistant subpopulations within bacterial populations, thereby expanding the reservoir of resistance at the population level [6,8].

The knowledge gaps and misconceptions consistently observed in university and community samples support the notion that antibiotic use behaviors have a direct impact on biological outcomes, notably the emergence and dissemination of resistant strains [7,9]. The sustainability of antimicrobial stewardship (AMS) approaches in the fight against AMR depends on the knowledge, attitudes, and practices (KAP) of the general public, particularly those of young adults. University students represent a critical group in this regard, as they both reflect current societal behaviors and influence future health literacy and clinical decision-making processes [7,10]. Studies examining differences between health sciences students and non-health sciences students have reported higher levels of clinical competence in health sciences students; however, even in these same studies, it has been observed that incorrect practices have not been completely eliminated [8,10]. Similarly, reports of moderate knowledge-attitude profiles and high self-medication rates among non-healthcare university students suggest that AMS interventions should not be limited to health professionals alone [6,9,11]. Northern Cyprus, where this study was conducted, is a high-risk epidemiological region in terms of antimicrobial resistance (AMR) due to relatively lax legal controls on the over-the-counter sale of antibiotics and the island’s generally high rates of antibiotic consumption [12,13]. Therefore, examining the behavioral patterns of young adults in such a critical region will not only fill a theoretical gap in the literature but also provide an evidence-based strategic foundation for strengthening regional public health policies and regulatory mechanisms.

However, many KAP studies in the literature are conducted at a descriptive level and are limited to studies that compare students’ knowledge levels based on demographic characteristics. Nevertheless, there remains a need to develop evidence-based predictive models to determine the extent to which cognitive deficits and inappropriate attitudes predict the risk of treatment nonadherence, which triggers the selection of resistant mutants in real-world data.

To prevent conceptual ambiguity and maintain methodological consistency throughout the study, the behavioral metrics are structured within a nested hierarchy. ‘Inappropriate antibiotic use’ serves as the overarching broad concept encompassing any irrational drug consumption (e.g., self-medication or viral misuse). Within this, ‘antimicrobial non-adherence’ is classified as a specific subset involving the failure to comply with a prescribed medical regimen. Finally, ‘premature discontinuation’ is operationally defined as the exact behavioral manifestation of non-adherence investigated in our predictive model, where a patient terminates their antibiotic course earlier than recommended based on subjective symptom improvement.

The aim of this study is to assess the levels of knowledge, attitudes, and practices regarding antibiotic use and resistance among a broad cohort of university students enrolled in nursing programs and other programs outside the field of health education. Going beyond descriptive studies in the literature, this study aims to demonstrate, through a multivariate model, how gaps in knowledge regarding pathogen-drug interactions and irresponsible attitudes predict treatment non-adherence, discussing the theoretical microbiological implications of these behaviors.

## 2. Results

### 2.1. Sociodemographic Characteristics and Data Distribution

The normality of the data was examined using the Kolmogorov–Smirnov and Shapiro–Wilk tests, and it was determined that the data were not normally distributed (*p* < 0.05). The overall internal consistency of the scale was calculated using Cronbach’s Alpha, and \alpha was found to be 0.806.

The sociodemographic characteristics of the study participants (*N* = 1044) are presented in Table 1. Most of the participants were female (64.6%, *n* = 674), while males accounted for 34.8% (*n* = 363) of the sample. Regarding academic distribution, 59.4% *(n* = 620) of the students were enrolled in the Nursing Department, and 40.6% (*n* = 424) were from other non-healthcare departments.

The distribution across academic years showed that first-year students constituted the largest group (34.1%, n = 356), followed by fourth-year (27.7%, n = 289), second-year (22.7%, n = 237), and third-year students (15.5%, n = 162). The mean age of the 1,044 students participating in the study was 22.12 ± 3.41. The demographic characteristics of the participants are presented in Table 1.

### 2.2. Comparison of KAP Scores by Department

The comparison of Knowledge, Attitude, and Practice (KAP) scores between nursing students and students from other (non-healthcare) departments is presented in Table 2.

The results of the Mann–Whitney U test revealed that nursing students achieved significantly higher median scores in all three dimensions compared to their counterparts (*p* < 0.001 for all dimensions).

Specifically, the median knowledge score for nursing students was 9 (IQR: 8–11), whereas students from other departments had a median score of 7 (IQR: 5–8) (*Z* = −6.421, *p* < 0.001). A similar trend was observed in the attitude scores, where nursing students demonstrated a median of 10 (9–11) compared to 8 (6–9) for the other group (*Z* = −5.232, *p* < 0.001). Furthermore, the median practice score was significantly higher among nursing students (median = 9, IQR = 8–10]) than among non-healthcare students (median = 7, IQR = 6–8; *Z* = −5.454, *p* < 0.001). These findings indicate that academic exposure to nursing education significantly enhances students’ antimicrobial awareness and fosters more responsible antibiotic-related behaviors.

To evaluate the longitudinal impact of nursing education, an intra-group analysis was performed specifically for nursing students across their academic years using the Kruskal–Wallis test. A statistically significant increase was observed from the 1st to the 4th year in all dimensions: Knowledge (*p* = 0.007), Attitude (*p* = 0.028), and Practice (*p* = 0.028). Specifically, the mean rank for knowledge scores among nursing students rose from 296 in the 1st year to 341 in the 4th year. This linear progression confirms the significant positive impact of the nursing curriculum on microbiological maturation and the development of professional antimicrobial awareness.

### 2.3. Correlation Analysis Between KAP Dimensions

The relationships between Knowledge, Attitude, and Practice (KAP) scores were evaluated using Spearman’s rank correlation coefficient (Table 3).

### 2.4. Predictors of Antimicrobial Non-Adherence

To identify the independent risk factors predicting antibiotic non-adherence, a multivariate logistic regression model was constructed (N = 1044). Before constructing the model, the assumptions of multicollinearity were tested; all Variance Inflation Factor (VIF) values were found to be between 1.01 and 1.07 (VIF < 10), and tolerance values were >0.1, confirming no significant multicollinearity between independent variables. Furthermore, the Hosmer-Lemeshow test confirmed an excellent model fit (*p* > 0.05) (Table 4).

**Figure 1:**
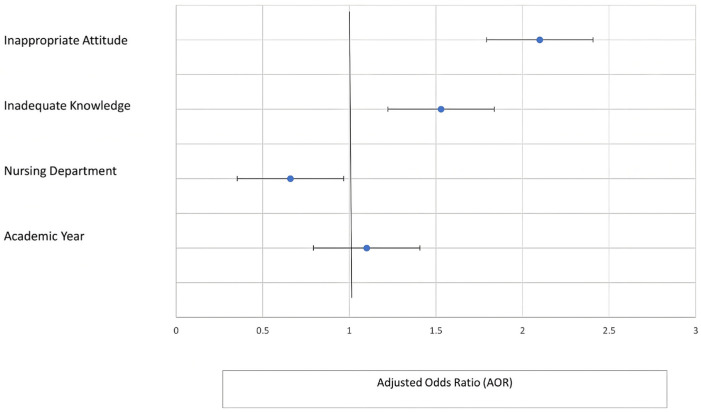
Forest plot of the multivariate logistic regression model identifying predictors of antimicrobial non-adherence (*N* = 1044). The vertical line at AOR = 1.0 represents the null hypothesis (no effect). Interpretation Guide: An Adjusted Odds Ratio (AOR) > 1.0 indicates an increased probability/risk of experiencing treatment non-adherence, whereas an AOR < 1.0 indicates a significant protective factor. Reference Categories: The reference baseline for the Department variable is ‘Other (Non-healthcare) departments’. The Academic Year variable is treated as a continuous linear predictor in the model. As illustrated, inappropriate attitudes (AOR: 2.100) and inadequate knowledge (AOR: 1.536) serve as independent risk factors, while nursing education (AOR: 0.669) operates as a significant protective barrier.

The final regression model revealed several significant predictors of antimicrobial non-adherence (Figure 1):

- Inappropriate Attitude: This was identified as the strongest independent predictor. Students with suboptimal or incorrect attitudes toward antibiotic use were 2.1 times more likely to exhibit non-adherent behaviors, such as premature cessation of treatment (AOR: 2.100; 95% CI: 1.586–2.780; *p* < 0.001).
- Inadequate Knowledge: Participants with a lack of sufficient microbiological knowledge had a 1.5-fold increase in the odds of treatment non-adherence (AOR: 1.536; 95% CI: 1.160–2.033; *p* = 0.003).
- Department (Nursing vs. Others): Enrollment in the nursing department was identified as a significant protective factor, reducing the likelihood of non-adherence by approximately 33% compared to non-healthcare students (AOR: 0.669; 95% CI: 0.504–0.888; *p* = 0.005).
- Academic Year: Interestingly, the student’s academic year did not emerge as a significant independent predictor of non-adherence in the final multivariate model (AOR: 1.108; 95% CI: 0.988–1.242; *p* = 0.079).

## 3. Discussion

Antimicrobial resistance (AMR) is a global public health crisis that threatens modern medicine’s ability to treat infections and, according to 2019 data, directly caused 1.27 million deaths. At the root of this crisis lies the anthropogenic selective pressure exerted on bacterial populations as a result of the inappropriate use of antibiotics. This study, which examined the knowledge, attitude, and practice (KAP) levels of university students (n = 1044), highlights the critical role not only of theoretical knowledge but also of the transformation of this knowledge into a professional attitude in preventing the selection of resistant mutants.

According to the findings of our study, nursing students’ scores regarding knowledge, attitudes, and practices related to antibiotic use and resistance were found to be statistically significantly higher (*p* < 0.001) than those of students in fields outside the health sciences.

This clear superiority in nursing students’ median scores for knowledge (9 vs. 7), attitude (10 vs. 8), and practice (9 vs. 7) strongly aligns with the findings of [9,10,14], which indicate that health sciences students have higher AMR awareness.

From a microbiological perspective, this situation demonstrates that nursing students, by understanding the difference between the type of pathogen (virus or bacterium) and the mechanism of antibiotic action, have the capacity to prevent the creation of unnecessary evolutionary pressure on commensal bacteria in the host’s normal microbiota and to mitigate the risk of potential horizontal gene transfer.

When nursing students were evaluated within their own group, the statistically significant increase observed in knowledge (*p* = 0.007), attitude (*p* = 0.028), and practice (*p* = 0.028) scores as the academic year progressed (from the first to the fourth year) is one of the most important findings of our study. This linear increase between the academic year and KAP scores supports the findings of the studies conducted by researchers [10,15].

This result demonstrates that a health-focused curriculum successfully prepares students to serve as clinical stewards who will help control the spread of resistant strains in hospital settings in the future.

Our correlation analysis of KAP dimensions sheds light on the dynamics of behavioral change. Our analyses revealed that the positive relationship between attitude and practice (*r* = 0.528, *p* < 0.001) is significantly stronger than the relationship between knowledge and practice (*r* = 0.357, *p* < 0.001). As emphasized in a study conducted in Israel [8], attitudes are a far more powerful predictor of human behavior than mere knowledge. This demonstrates that theoretical knowledge of microbiology alone is insufficient in the fight against AMR, and that professional and ethical attitudes serve as a key bridge in the process of translating knowledge into practice.

The most striking and microbiologically critical finding of our study was obtained from the multivariate logistic regression model predicting non-adherence to antibiotic therapy (premature discontinuation of medication). Our model demonstrated that the strongest independent risk factor triggering inappropriate antibiotic use was incorrect attitude (AOR: 2.100, *p* < 0.001), followed by insufficient microbiological knowledge (AOR: 1.536, *p* = 0.003).

When patients stop their antibiotic treatment as soon as they feel better, this exposes the bacterial population to sub-lethal concentrations (below the lethal dose). Insufficient exposure to the drug eliminates susceptible strains while allowing mutants with low-level resistance to survive and become selectively enriched. The fact that individuals lacking knowledge and awareness face this risk approximately 1.5 to 2 times more frequently demonstrates that AMR is not merely a genetic adaptation process but also an evolutionary process accelerated by inappropriate human behavior.

The finding that incorrect attitudes (AOR: 2.100) are a stronger predictor of treatment non-adherence than insufficient information (AOR: 1.536) is strongly consistent with the current international literature. For example, researchers [16,17] have emphasized that non-adherence behaviors, such as premature discontinuation of antimicrobial agents and changes in dosage, are generally driven not so much by patients’ overt (obvious) lack of information, but rather by the personal health beliefs and attitudes they develop regarding the necessity of the medication and the treatment process. Our study demonstrates that, in the process of translating microbiological knowledge into clinical practice, personal attitudes constitute the strongest psychological barrier to reducing selective pressure, in line with similar findings in Asian and Middle Eastern populations [18].

This critical nexus highlights a ‘Behavioral Microbiology’ perspective: even when individuals possess theoretical knowledge, their subjective ‘health beliefs’ override their cognitive understanding, directly managing the biological outcome. This behavioral pattern leads to sub-lethal dose exposure; discontinuing treatment based on perceived recovery theoretically risks creating a selective window that, according to established microbiological literature, is associated with the survival and enrichment of resistant mutants. Therefore, public health models should evaluate AMR dynamics alongside these specific psychological decision-making patterns that theoretically influence sub-lethal dose exposure risk [19].

On the other hand, the fact that our model identified studying in the nursing department as a significant protective factor that reduces the likelihood of engaging in this risky behavior (AOR: 0.669, *p* = 0.005) underscores just how powerful a barrier microbiology education is in protecting public health.

Our findings provide epidemiological support for the notion—increasingly emphasized in the modern literature—that nurses in AMS programs are not merely medication administrators; they serve as antibiotic first responders in processes such as infection control, culture monitoring, and the intravenous-to-oral (IV to PO) switch [20,21].

Conversely, the fact that students enrolled in non-medical faculties are at high risk for non-adherence to treatment and inappropriate antibiotic use presents a global challenge. A recent KAP study conducted in Saudi Arabia reported that, due to their lack of formal pharmacology education, non-medical students discontinue antibiotic treatment prematurely as soon as they feel clinically better and increasingly use leftover antibiotics [22]. The high-risk profile of non-nursing departments in our findings is directly consistent with other university cohorts based in Ethiopia and China [23,24,25], which indicate that these groups do not fully grasp the irreversible public health consequences of antimicrobial resistance.

## 4. Materials and Methods

### 4.1. Study Design and Setting

This descriptive and cross-sectional study was conducted at a university in Northern Cyprus between June 2025 and January 2026. The study was specifically designed as a KAP (Knowledge, Attitude, and Practice) survey to evaluate the impact of nursing education on antimicrobial awareness in an epidemiological setting with high antibiotic consumption rates.

### 4.2. Participants and Sampling

The study utilized a non-probability purposive sampling method to recruit participants from two distinct cohorts within the institution and was designed to analyze the protective barrier effect of specialized healthcare education against inappropriate antibiotic use. The university has a total student population of approximately 12,000. Participants were categorized as follows:

- Nursing Group (Positive Control Group): All students enrolled in the Department of Nursing (*N* = 888) were targeted for participation to represent a cohort with formal clinical and microbiological training. This group serves as a benchmark for high health literacy and professional accountability in antimicrobial stewardship (AMS). A total of 620 nursing students provided complete responses, yielding a high response rate of 69.8%.
- Non-Healthcare Group (General Population Proxy): A comparison group of 424 students was recruited from departments that do not provide health or medical curricula (e.g., engineering, business, social sciences). This cohort represents the general young adult population and allows for the identification of behavioral risks in the absence of formal medical education.

Exclusion Criteria: To isolate the impact of nursing-specific education, students from other medical and health-related fields—including Medicine, Dentistry, and Pharmacy (comprising approximately 2500 students)—were excluded from the study.

### 4.3. Sample Size Calculation

The minimum required sample size was determined using the Yamane (Slovin) formula for a finite population (N\approx. 12,000):

$$n=\frac{N}{1+N{\left(e\right)}^{2}}$$

Based on a 95% confidence level and a 5% margin of error (*e* = 0.05), the minimum required sample size was calculated as 387. The final sample of 1044 participants significantly exceeded this requirement, ensuring high statistical power for multivariate analysis.

### 4.4. Data Collection Instrument

Data were collected via an online, structured self-administered questionnaire (Google Forms). The survey instrument was developed through an extensive synthesis of validated items previously employed in studies conducted in China [23], Saudi Arabia [26], and South Korea [27]. These specific sources were selected to ensure the instrument’s clinical relevance and cross-cultural validity regarding antibiotic use and public health behaviors. This integrative approach informed the structural development of the questionnaire, which was organized into four distinct sections to capture a comprehensive profile of the participants’ perspectives.

Socio-demographics (10 items): Gathered data on gender, age, country, academic department, academic year, chronic diseases, immune system status, smoking habits, and past-year infection/antibiotic experience.

Knowledge (11 items): Evaluated understanding of bacterial vs. viral infections, antibiotic effectiveness, side effects, and resistance development using dichotomous (Correct/Incorrect) scoring.

Attitude (11 items): Assessed professional responsibility, perceptions of the global AMR threat, and beliefs regarding over-the-counter access through clinical and ethical scenarios (Agree, Disagree, Neither agree nor disagree).

Practice (11 items): Focused on self-medication habits, treatment adherence (e.g., non-completion of prescribed courses), and home-stocking of antibiotics (Agree, Disagree, Neither agree nor disagree).

### 4.5. Validity and Reliability

The content and face validity of the KAP questionnaire were rigorously assessed by a panel of independent experts in clinical microbiology and public health. This process ensured that the 11 items in each section accurately represented the current microbiological and clinical guidelines regarding antibiotic resistance and usage. The overall internal consistency of the scale was confirmed with a Cronbach’s Alpha of 0.806.

### 4.6. Ethical Considerations

The study was conducted in accordance with the Declaration of Helsinki. Ethical approval was granted by the Cyprus International University Institutional Scientific Research and Publication Ethics Committee on 12 June 2025 (Decision Number: EKK24-25/13/09). All participants provided digital informed consent before participating in the survey.

### 4.7. Statistical Analysis

Statistical analyses were performed using IBM SPSS Statistics (Version 26.0, IBM Corp., Armonk, NY, USA). Normality was assessed using Kolmogorov–Smirnov and Shapiro–Wilk tests; since data were not normally distributed (*p* < 0.05), non-parametric tests (Mann–Whitney U and Kruskal–Wallis) were utilized for comparisons. Relationships between KAP dimensions were evaluated using Spearman’s rank correlation. A multivariate logistic regression model was constructed to identify independent predictors (Adjusted Odds Ratio [AOR]) of antibiotic non-adherence. Model fit was confirmed with the Hosmer-Lemeshow test (*p* > 0.05), and multicollinearity was checked via VIF values (<10). The categorical sample size variance between the Nursing (*n* = 620) and non-healthcare (*n* = 424) groups was mathematically accounted for by utilizing these non-parametric and multivariate logistic regression frameworks, which maintain statistical power and resilience under unequal sample sizes.

During the preparation of this work the authors used NotebookLM, DeepL, and Gemini to synthesize literature during the introduction phase, translate and refine the manuscript’s language, and conduct final reference consistency checks. After using these tools, the authors reviewed and edited the content as needed and take full responsibility for the content of the published article.

#### Operationalization and Definition of the Outcome Variable

To build a reproducible predictive framework, ‘antimicrobial non-adherence’ was operationalized as a binary outcome variable based on the critical practice statement ‘U1: I stop taking antibiotics when I start feeling better’. Following the methodological frameworks established by Almomani et al. (2022) [16] for short-term antibiotic adherence assessment, responses were dichotomized into a binary event matrix: hazardous non-adherent clinical practice (agreement with dropping medication prematurely) was coded as ‘1’ (Risk Event), while rational adherence (completing the prescribed therapeutic course) was designated as ‘0’ (Reference Baseline). The deployment of this specific item and its categorical cutoff was adapted from international validated KAP instruments previously verified in university student populations by Huang et al. (2013) [23] and Alnasser et al. (2021) [26], ensuring both content validity and cross-study comparison metrics.

### 4.8. Limitations

While this study provides strong multivariate evidence regarding the behavioral predictors of antibiotic non-adherence among a large university cohort (*N* = 1044), certain limitations must be acknowledged. First, our data relies entirely on cross-sectional, self-reported surveys, which may introduce recall or social desirability biases. Second, substantial gender imbalance was observed, with female participants accounting for 64.6% of the sample. This distribution reflects the natural demographic reality of the targeted nursing student population, which is historically female-dominated in the region; however, it should be considered when generalizing the descriptive gender trends. Third, this study did not include real-time microbiological tracking, laboratory resistance surveillance, minimum inhibitory concentration (MIC) measurements, or longitudinal adherence monitoring. Therefore, the links discussed between non-adherence behaviors and resistant mutant selection are based on established theoretical mechanisms in the literature rather than direct experimental outcomes of this research. Future studies combining behavioral KAP tools with direct microbiological screening are warranted to validate these epidemiological connections empirically.

## 5. Conclusions

This study demonstrates that public risk factors surrounding antibiotic resistance (AMR) are closely tied to human behavioral management and perceptual errors, which can act as behavioral catalysts in potential resistance dynamics. Findings from our logistic regression model indicate that incorrect attitudes and insufficient microbiological knowledge are key risk factors driving individuals to discontinue treatment prematurely (non-adherence). From a conceptual standpoint, such non-adherent behaviors are highly plausible contributors to selective pressure, exposing bacterial populations to sublethal concentrations and theoretically expanding the resistance reservoir at the population level.

In conclusion, our multivariate model confirms that the fight against AMR is not solely a pharmacological or clinical issue but a complex process of psychological behavior management. While nursing education serves as a significant protective factor (AOR: 0.669), the high risk identified in non-healthcare students underscores the need for behavioral-focused interventions. Future antimicrobial stewardship (AMS) programs must transcend traditional ‘information-giving’ models and incorporate behavioral economics principles to modify the ingrained attitudes that trigger resistant mutant selection.

Particularly in regions such as Northern Cyprus, where access to over-the-counter antibiotics is relatively unrestricted, the following technology-driven strategic policies are recommended to proactively manage these biological risks, going beyond traditional approaches:

Evidence-Based Diagnosis and POCT Integration: Rapid antigen tests (POCT) should be used at the pharmacy level to enable on-site differentiation between viral and bacterial infections; this will help minimize the unnecessary evolutionary pressure that empirical antibiotic use places on the normal microbiota. Digital Nudging Systems to Improve Adherence: Mobile apps developed based on behavioral economics principles should remind patients of the microbiological consequences of discontinuing treatment and ensure they complete the full course of medication beyond the MIC (Minimum Inhibitory Concentration).

Interactive Digital Health Literacy and Patient Engagement Modules Based on JCI Standards: Evidence-based and standardized digital education platforms that are fully compliant with JCI’s Patient and Family Education (PFE) standards should be implemented. These modules, which bring the shared decision-making process between physician and patient onto a technological platform, should interactively visualize the specific effects of the antibiotic on pathogen-host interactions and the pharmacokinetic/microbiological (resistance) risks associated with prematurely discontinuing treatment, rather than relying on traditional passive materials. Furthermore, in accordance with the JCI principle of verifying understanding of education—a critical requirement—these e-Health tools must assess the patient’s cognitive level through integrated teach-back mechanisms; thereby transforming the patient from a passive recipient into an active partner who maintains explicit accountability in treatment compliance and medication safety (IPSG 3) processes.

Monitoring Resistance Hotspots with Artificial Intelligence: By analyzing pharmacy sales data in real time using artificial intelligence algorithms, regional risks of resistant strain spread (resistance hotspots) should be mapped, and location-specific public health interventions should be initiated.

The Antimicrobial Stewardship Model Led by Nurses: Based on the high protective profile identified among nursing students, the goal should be to position this group as clinical stewards within the community and to reduce behavioral risks in non-healthcare fields through peer education models.

In conclusion, our logistic model has demonstrated that antibiotic resistance is not merely a biological problem developing in a laboratory setting, but rather an anthropogenic process driven by users’ attitudes and knowledge gaps. To prevent the selection of drug-resistant mutants, it is essential that higher education curricula not only provide theoretical knowledge of microbiology but also supplement this knowledge with practical training that translates it into a sense of clinical responsibility.

## Figures and Tables

**Table 1:** Sociodemographic characteristics of the study participants (*N* = 1044).

Characteristics	Frequency (n)	Percentage (%)
**Gender**		
Female	674	64.6
Male	363	34.8
Other	7	0.7
**Department**		
Nursing	620	59.4
Other (Non-healthcare)	424	40.6
**Academic Year**		
1st Year	356	34.1
2nd Year	237	22.7
3rd Year	162	15.5
4th Year	289	27.7
**Age (years)**	22.12 ± 3.41 *	

Note: Data for age is expressed as Mean ± Standard Deviation (SD). * Indicates that the variable exhibits a non-normal distribution profile (*p* < 0.05) based on Kolmogorov–Smirnov and Shapiro–Wilk normality testing.

**Table 2:** Comparison of Knowledge, Attitude, and Practice (KAP) scores according to the students’ departments.

KAP Dimensions	Nursing Students (*n* = 620) Median (IQR)	Other Students (*n* = 424) Median (IQR)	Mann–Whitney U (*Z*)	*p*-Value *
**Knowledge Score**	9 (8–11)	7 (5–8)	−6.421	**<0.001**
**Attitude Score**	10 (9–11)	8 (6–9)	−5.232	**<0.001**
**Practice Score**	9 (8–10)	7 (6–8)	−5.454	**<0.001**

Notes: * Mann–Whitney U test was applied for independent group comparisons. Bold values indicate strict statistical significance at the *p* < 0.05 level. Abbreviations: IQR: Interquartile Range; Z: Standardized Mann–Whitney U test statistic.

**Table 3:** Spearman’s rank correlation matrix between KAP dimensions.

Dimensions	1. Knowledge	2. Attitude	3. Practice
**1. Knowledge**	1.000		
**2. Attitude**	0.471 **	1.000	
**3. Practice**	0.357 **	0.528 **	1.000

** Correlation is significant at the 0.01 level (2-tailed).

**Table 4:** Final multivariate logistic regression model predicting the risk factors for antimicrobial non-adherence (N = 1044).

Independent Variables	β	Wald	*p*-Value	Adjusted OR (95% CI)
**Inappropriate Attitude**	0.742	26.827	**<0.001**	2.100 (1.586–2.780)
**Inadequate Knowledge**	0.429	8.985	**0.003**	1.536 (1.160–2.033)
**Department** (Nursing vs. Others *)	−0.403	7.747	**0.005**	0.669 (0.504–0.888)
**Academic Year** (Class)	0.102	3.076	0.079	1.108 (0.988–1.242)
**Constant**	0.104	0.154	0.695	1.109

* Reference category for department is “Other (Non-healthcare) departments”. CI: Confidence Interval, OR: Odds Ratio. Bold values indicate statistical significance (*p* < 0.05). Model Fit: Hosmer-Lemeshow test (*p* > 0.05); Multicollinearity was checked, and all VIF values were <10. Note: T8 refers to the misconception that antibiotics treat colds/coughs; B2 refers to the lack of knowledge regarding viral infections.

## Data Availability

The original contributions presented in this study are included in the article/Supplementary Material. Further inquiries can be directed to the corresponding author.

## Supplementary Materials

The following supporting information can be downloaded at: https://www.mdpi.com/article/10.3390/antibiotics15060569/s1.
